# A Case of Borderline Tuberculoid Leprosy With Type One Lepra Reaction

**DOI:** 10.7759/cureus.38081

**Published:** 2023-04-24

**Authors:** Ganaraja V Harikrishna, Jayashankar CA, Sravya Doddapaneni, Nuthan Bhat, Prakash Bhanu

**Affiliations:** 1 Neurology, Vydehi Institute of Medical Sciences and Research Centre, Bangalore, IND; 2 Internal Medicine, Vydehi Institute of Medical Sciences and Research Centre, Bangalore, IND; 3 Dermatology, Vydehi Institute of Medical Sciences and Research Centre, Bangalore, IND

**Keywords:** type one lepra reaction, mycobacterium leprae, reversal reaction, unilateral facial nerve palsy, borderline tuberculoid leprosy

## Abstract

Leprosy is a chronic infectious disease caused by *Mycobacterium leprae*, which primarily affects the skin and peripheral nerves. The variants that can be identified include tuberculoid (TT), borderline tuberculoid (BT), mid-borderline (BB), borderline lepromatous (BL), and lepromatous forms (LL). Type one lepra reactions are delayed hypersensitivity reactions that are often observed in borderline variants due to an unstable immunological response. They can exacerbate skin lesions and neuritis, leading to a higher risk of disabilities and deformities. Early detection and management would play a major role in limiting morbidity. Here, we present a case of a 46-year-old male diagnosed with borderline tuberculoid leprosy on multidrug therapy who developed features suggestive of type one lepra reaction. Early recognition of this entity helps in mitigating the risk of permanent nerve damage, disability, deformity, and morbidity.

## Introduction

Leprosy is a complex infectious disease with primarily dermatological and neurological manifestations with the highest incidence in India, Brazil, and Indonesia [1]. Droplet infection through nasal mucosa is widely accepted as the most important mode of transmission [1]. It is classified based on Ridley-Jopling classification into five main variants based on clinical and histopathological picture [2]. They include tuberculoid (TT), borderline tuberculoid (BT), mid-borderline (BB), borderline lepromatous (BL), and lepromatous forms (LL) [2].

Borderline variants are associated with type one lepra reactions in around 30% of the patients often within 12 months of treatment initiation [1]. Possible manifestations include acute painful neuritis, ulcers, loss of neurological function, thickening of nerves, abscesses, high fever, and sudden urticaria of leprotic skin lesions, which occur due to increased inflammation of preexisting lesions [1,3]. These reactions when diagnosed and treated at the earliest can limit further nerve damage and loss of neurological function [4]. Hence, it is essential for clinicians to be vigilant about the different manifestations of type one lepra reactions occurring in association with borderline forms of the disease.

In this report, we present a case of a middle-aged male patient with preexisting borderline tuberculoid leprosy on a multidrug regimen with type one lepra reaction and lower motor neuron unilateral facial nerve palsy.

## Case presentation

A 46-year-old male diagnosed with borderline tuberculoid leprosy 14 months ago with the help of a slit skin smear on multidrug therapy presented to our tertiary care hospital with complaints of a solitary ulcer over the left thumb and recurrent ulcers over bilateral lower limbs. In addition, the patient complained of involuntary movements of the left hand, tingling sensation over the face and trunk, inability to close the eyes with frequent lacrimation, intermittent fever, and episodes of epistaxis over the past six months. The patient denied changes in weight, trauma prior to the occurrence of ulceration, hoarseness of voice, and occurrence of other skin lesions. He was compliant with his multidrug regimen for the treatment of leprosy and was also taking oral amitriptyline, oral pregabalin, and multivitamin supplements.

**Figure 1:**
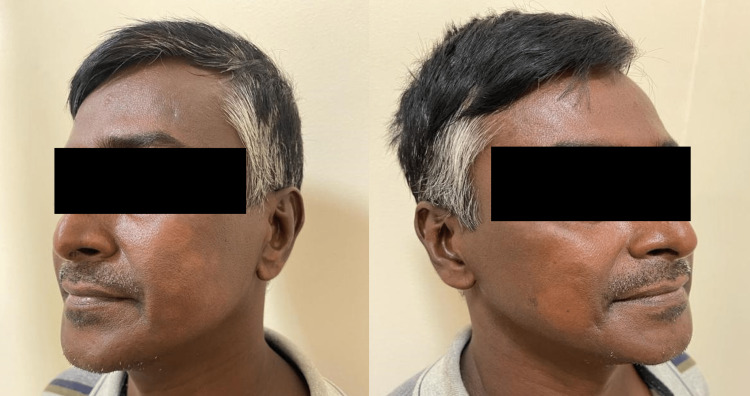
Multiple well-defined hypopigmented patches over bilateral cheeks.

**Figure 2:**
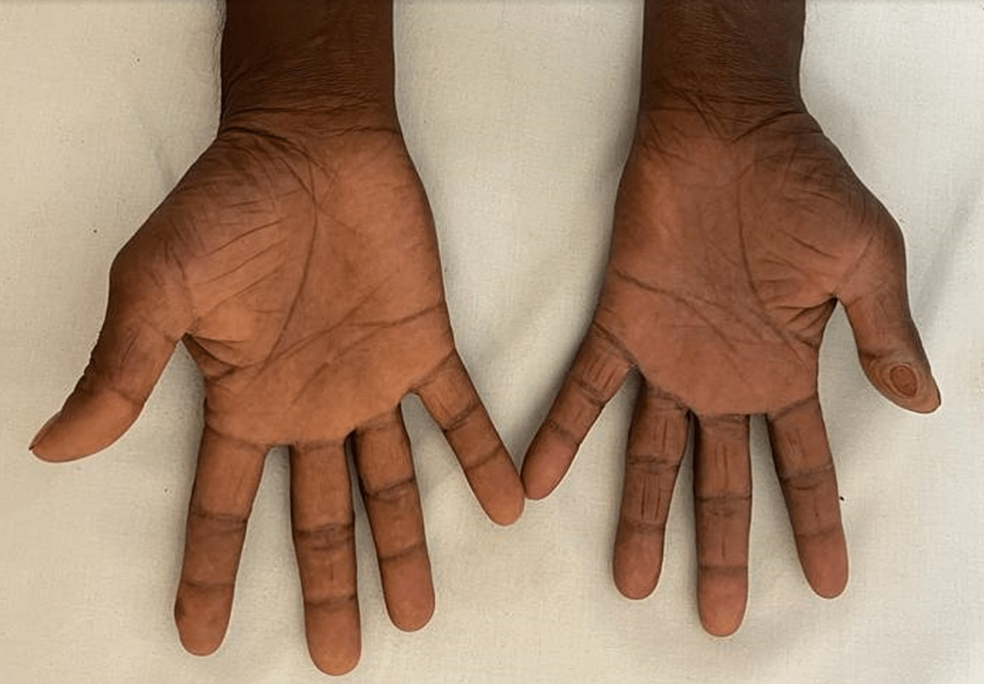
A solitary well-defined ulcer with an erythematous base over the palmar aspect of the left thumb measuring 2×2 centimeters.

**Figure 3:**
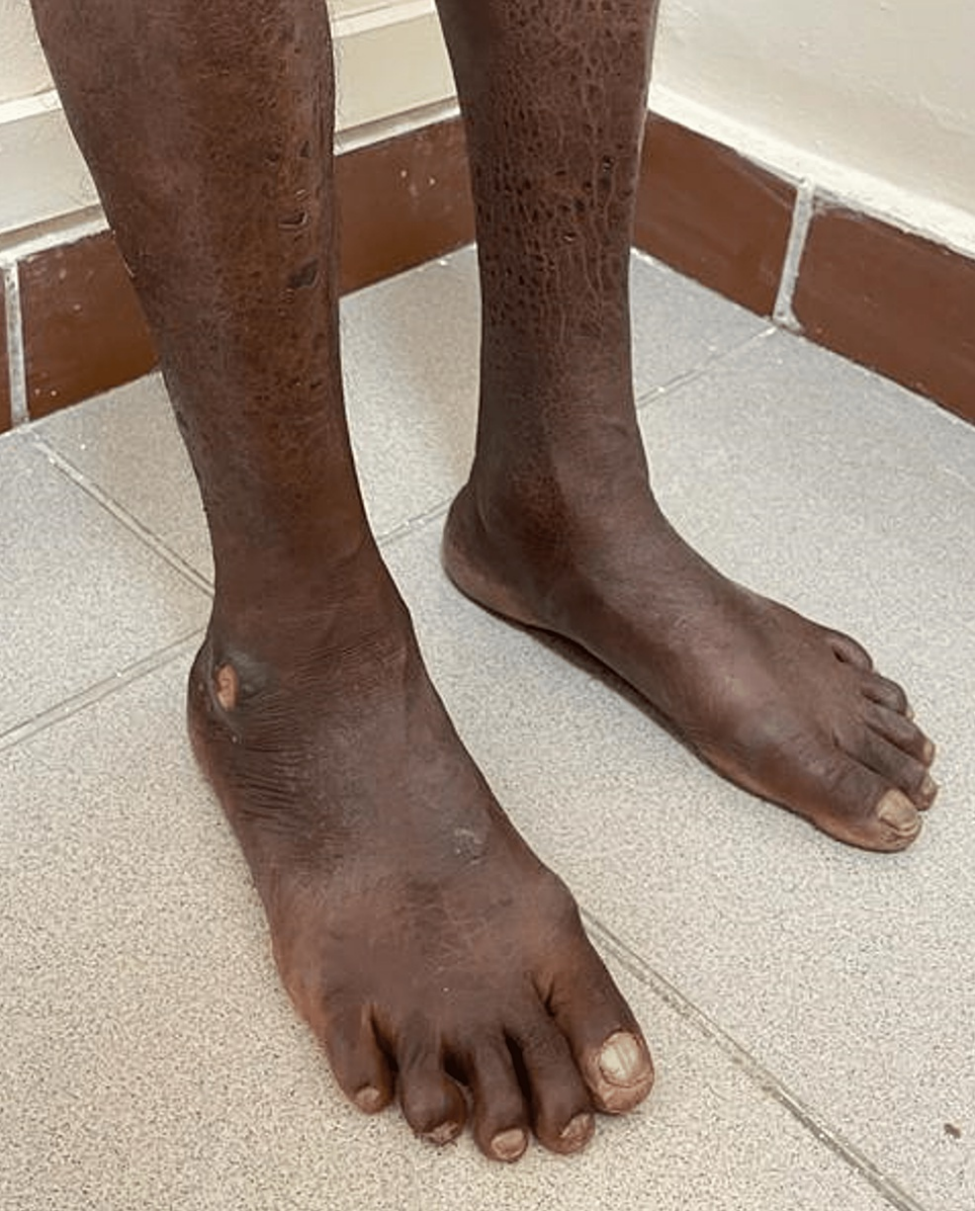
A solitary well-defined punched-out non-healing ulcer over the right lateral malleolus measuring 3×2 centimeters.

Local examination confirmed the presence of borderline tuberculoid leprosy with multiple well-defined hypopigmented patches over bilateral cheeks, the largest measuring around 4×4 centimeters and the smallest measuring around 1×2 centimeters (Figure 1), and multiple ichthyotic scaling over bilateral lower limbs. A solitary well-defined ulcer with an erythematous base was observed over the palmar aspect of the left thumb measuring 2×2 centimeters (Figure 2). A solitary well-defined punched-out ulcer was observed over the right lateral malleolus measuring 3×2 centimeters (Figure 3).

**Figure 4:**
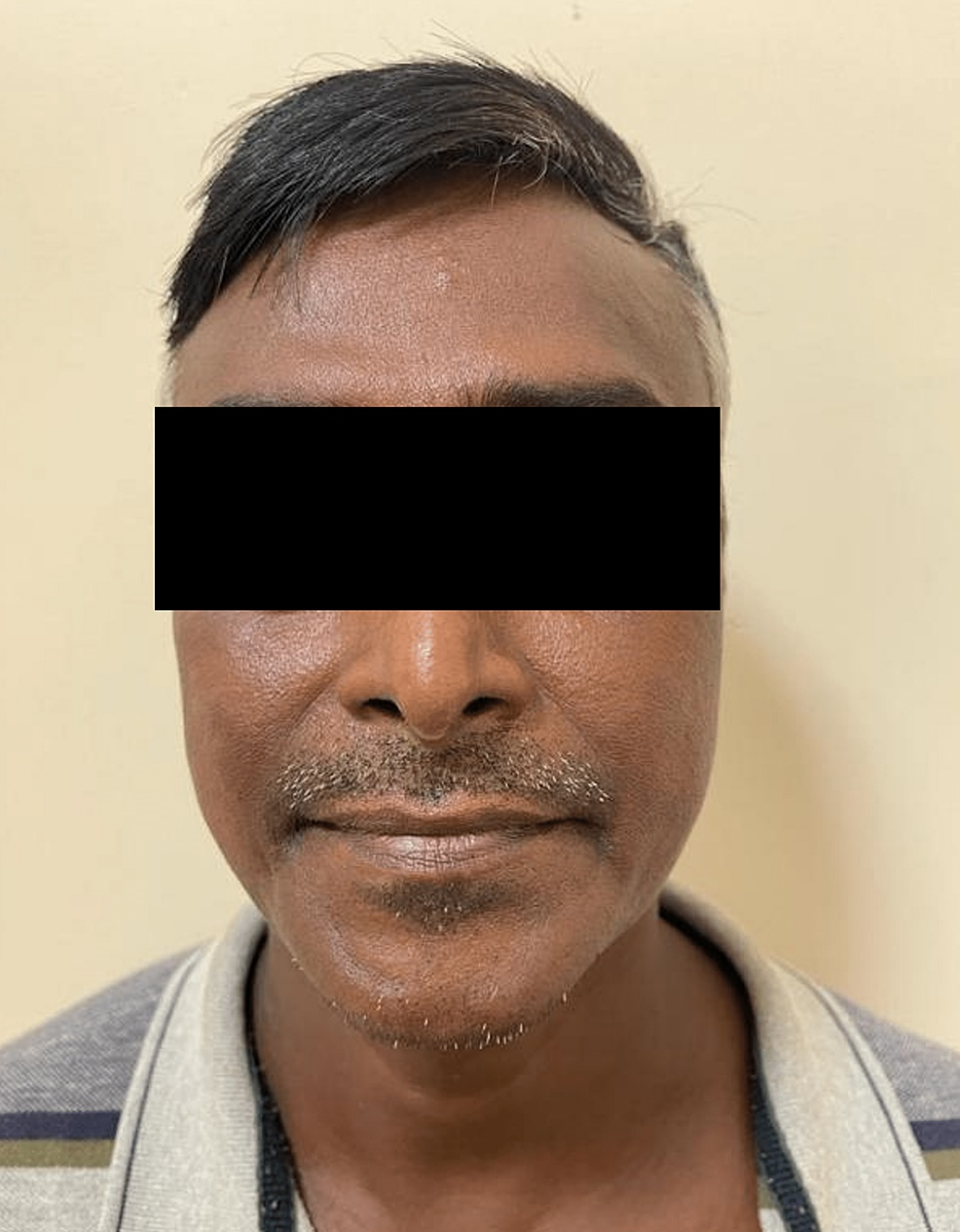
Reduced wrinkles over the right forehead and flattening of the right nasolabial fold suggestive of lower motor neuron type right facial nerve palsy.

On nervous system examination, the left and right supraorbital, right greater auricular, left ulnar, and left common peroneal nerves were thickened. Loss of sensation was observed over the left ulnar nerve distribution and over the left foot. On motor examination, a weak left-hand grip was noted with weak left-sided reflexes. On cranial nerve examination, incomplete closure of the right eye was observed. There were reduced wrinkles over the right forehead and flattening of the right nasolabial fold suggestive of lower motor neuron type right facial nerve palsy (Figure 4). The cardiovascular, pulmonary, and gastrointestinal examination was unremarkable.

An initial slit skin smear from an affected lesion was positive for acid-fast bacilli. An edge-wedge biopsy from the active margins of the lesions performed at the time showed features suggestive of borderline tuberculoid leprosy and type one lepra reaction. The patient was commenced on a daily dose of oral prednisolone of 40 milligrams, which was gradually tapered off to a maintenance dose of 10 milligrams daily for a duration of 12 weeks in addition to his preexisting multidrug regimen for leprosy. The patient responded well to the steroids and was symptomatically better at the end of the course of prednisolone. He was also advised to use moxifloxacin eye drops and carboxymethylcellulose eye drops to prevent dryness and infection of the eyes due to incomplete closure.

**Figure 5:**
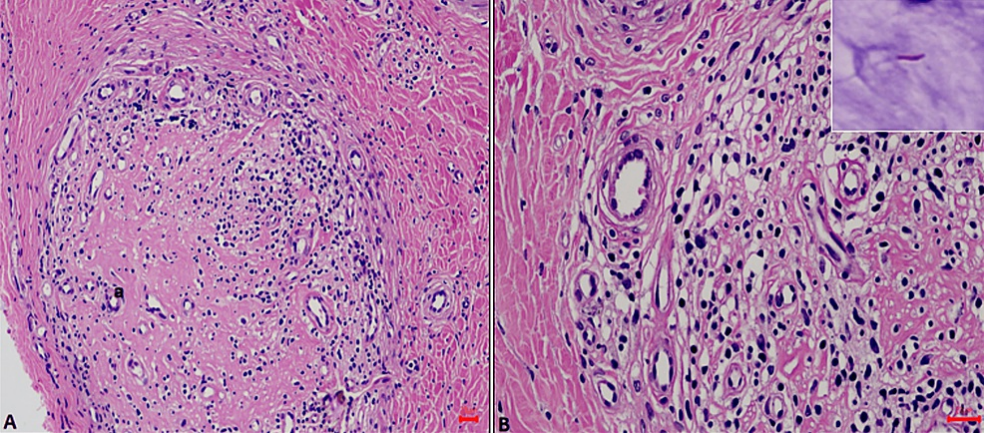
A: Hematoxylin and eosin-stained section showing prominent lymphocytic inflammation in the epineurium, perineurium, and endoneurium. The endoneurium and epineurium were densely hyalinized. There were no granulomas. B: Several foam cells and occasional lepra bacilli were seen (inset: Fite Faraco stain) (scale bars represent 20 microns).

A repeat slit skin smear after four months showed no acid-fast bacilli. Nerve conduction studies showed asymmetrical sensorimotor axonal peripheral neuropathy worse on the left limbs. A nerve biopsy from the sural nerve marked lymphoplasmacytic infiltrate in all three compartments: epineurium, perineurium, and endoneurium. There was marked epineurial and endoneurial collagenization and hyalinization with marked thickening of the perineurium. The epineurial nutrient artery showed thickening of the wall, subintimal fibrosis, and narrowing of the lumen. Fite Faraco stain revealed occasional lepra bacilli. These findings were suggestive of borderline tuberculoid leprosy, hence confirming a diagnosis of type one lepra reaction (Figure 5).

The patient was recommended to stay adherent to the multidrug treatment consisting of oral rifampicin, clofazimine, and dapsone. Ulcer debridement followed by topical mupirocin ointment and dressing was recommended biweekly for wound management.

## Discussion

Leprosy is a chronic infectious disease that primarily affects the skin and peripheral nerves. It is caused by *Mycobacterium leprae* most importantly through droplet transmission via nasal mucosa [1]. Its clinical manifestations and course are dependent on host immunity, exposure, and bacterial load, with a large percentage of affected patients undergoing spontaneous resolution [1]. Classification of leprosy is essential to determine treatment, risk of complications, and prognosis [5]. According to the Ridley-Jopling classification, five variants have been described: tuberculoid, borderline tuberculoid, mid-borderline, borderline lepromatous, and lepromatous forms [2].

Leprosy reactions, also termed reversal reactions, pose a significant challenge in the management of leprosy due to their role in causing permanent disability and nerve damage [6]. They occur due to the variable immunological response to the causative bacteria and may occur before, during, or after the completion of multidrug therapy [6]. Their two major forms include type one and type two leprosy reactions. Type one lepra reactions are delayed hypersensitivity reactions that are observed to occur in borderline forms, whereas type two lepra reactions are more frequently seen in the lepromatous variant [6]. A type one reaction despite adherence to therapy is generally due to immunological upgrading associated with a simultaneous increase in cell-mediated immunity, hence representing a transition to the tuberculoid type [1]. Type one lepra reactions are characterized by acute inflammatory changes of preexisting lesions leading to neuritis, ulcers, loss of neurological function, thickening of nerves, abscesses, and high fever [1,3,6]. These reversal reactions can lead to permanent nerve damage, deformity, disability, and morbidity, making it imperative for clinicians to recognize them sooner and provide adequate treatment at the earliest [6].

The association between type one lepra reaction and borderline tuberculoid leprosy has been well-documented [4,7,8]. However, clinicians need to understand and visualize the various manifestations of type one leprosy reaction in different patients as it can present in a variable fashion each time. Hence, our case report and associated images seek to aid clinicians in the diagnosis of type one leprosy reactions and add to the database of related literature for the education of clinicians and medical students.

Type one lepra reactions are treated with corticosteroids that inhibit pro-inflammatory cytokines with doses that are slowly tapered over a period of three to six months [6]. Our patient was treated with a 12-week course of oral prednisolone with slow tapering in addition to regular debridement and mupirocin ointment over ulceration. Regular follow-up was recommended to optimize treatment and provide early rehabilitation to prevent and manage deformities and disabilities.

## Conclusions

Our case report describes a middle-aged male previously diagnosed with borderline tuberculoid leprosy on multidrug therapy who developed features suggestive of type one lepra reaction. Early treatment with oral prednisolone was initiated with regular debridement and application of mupirocin ointment over ulcers. Owing to the fact that nearly 30% of cases of borderline variants of leprosy are associated with type one lepra reaction, the clinician must be vigilant about its different manifestations and ensure early diagnosis and treatment. This helps in mitigating the risk of permanent nerve damage, disability, deformity, and morbidity.
